# Association between quality control and outcomes of septic shock caused by intestinal perforation in China: a cross-sectional study

**DOI:** 10.1038/s41598-023-30551-w

**Published:** 2023-02-27

**Authors:** Lu Wang, Xudong Ma, Huaiwu He, Longxiang Su, Yanhong Guo, Guangliang Shan, Ye Wang, Xiang Zhou, Dawei Liu, Yun Long, China-NCCQC group

**Affiliations:** 1 Department of Critical Care Medicine, State Key Laboratory of Complex Severe and Rare Diseases, Peking Union Medical College Hospital, Peking Union Medical College and Chinese Academy of Medical Sciences, Beijing, 100000 China; 2 Department of Medical Administration, National Health Commission of the People’s Republic of China, Beijing, 100000 China; 3 Department of Epidemiology and Biostatistics, Institute of Basic Medicine Sciences, Chinese Academy of Medical Sciences (CAMS) & School of Basic Medicine, Peking Union Medical College, Beijing, 100000 China

**Keywords:** Outcomes research, Trauma

## Abstract

Septic shock, largely caused by intestinal perforation, is a common critical disease in intensive care unit (ICU). For hospitals and health systems, a performance improvement program for sepsis was strong recommended in guidelines. Numerous studies have shown that improved quality control improves outcomes in patients with septic shock. Nevertheless, association between quality control and outcomes of septic shock caused by intestinal perforation are not fully revealed. Thus we designed this study to investigate effects of quality control on septic shock caused by intestinal perforation in China. This was a multicenter observational study. A total of 463 hospitals were enrolled in this survey, led by the China National Critical Care Quality Control Center (China-NCCQC) from January 1, 2018 to December 31, 2018. In this study, the indicators of quality control included the proportion of ICU patient bed occupancy to total inpatient bed occupancy, the proportion of ICU patients with APACHE II score ≥ 15, and the microbiology detection rate before antibiotic use. The outcome indicators included hospital stays, hospitalization costs, complications, and mortality. Generalized linear mixed models were used to analyse the association between quality control and septic shock caused by intestinal perforation. The proportion of ICU patient bed occupancy to total inpatient bed occupancy is positively correlated with hospital stays, incidence of complications (ARDS, AKI) and costs in septic shock caused by intestinal perforation (*p* < 0.05). The proportion of ICU patients with APACHE II score ≥ 15 was not associated with hospital stays and incidence of ARDS and AKI (*p* < 0.05). Increasing of the proportion of ICU patients with APACHE II score ≥ 15 decreased the costs of patients with septic shock caused by intestinal perforation (*p* < 0.05). The microbiology detection rate before antibiotic use was not associated with hospital stays, incidence of AKI and costs of patients with septic shock caused by intestinal perforation (*p* < 0.05). Surprisingly, the increase of microbiology detection rate before antibiotic use increased the incidence of ARDS in patients with septic shock caused by intestinal perforation (*p* < 0.05). The above three indicators of quality control were not associated with mortality of the patients with septic shock caused by intestinal perforation. On the one hand, the number of ICU patients admitted should be controlled to reduce the proportion of ICU patients out of total inpatient bed occupancy. On the other hand, intensive care unit admission of severe patients (patients with APACHE II score ≥ 15) should be encouraged to improve the proportion of patients with APACHE II score ≥ 15 in the ICU, so that ICU can focus more on the treatment of severe patients and promote the professionalization of severe patient management. It is not advisable to collect sputum specimens too frequently for patients without pneumonia.

## Introduction

Septic shock is a common critical disease in intensive care unit (ICU), with long hospital stay, many complications, high cost and high mortality^1,2^. In international guidelines for management of sepsis and septic shock 2021, a performance improvement program for sepsis was strong recommended for hospitals and health systems^3^. Intestinal perforation is one of the important causes of septic shock^4^. Numerous studies have shown that improved quality control improves outcomes in patients with septic shock. Septic shock caused by intestinal perforation differs significantly from other types of septic shock. The differences are mainly reflected in the following aspects. First, prognosis is more closely related to removal of the focus of infection and often requires surgical intervention. Second, the pathogenic bacteria of this type of septic shock mostly covers various intestinal bacteria and even fungi, and a variety of antibiotics need to be combined with each other. Third, the prognosis of this type of septic shock is often good after appropriate treatment. For these reasons, the prognosis of septic shock caused by intestinal perforation is more susceptible to quality control. There are few reports on effects of quality control on septic shock caused by intestinal perforation. Therefore, we designed this survey to specifically investigate effects of quality control on septic shock caused by intestinal perforation and to find a new way for the treatment of such patients.

## Methods

### Study design

This was a nationwide, observational database study in 2018. Hospitals were enrolled from the Quality Improvement of Critical Care Program (QICC Program), which was initiated by the China National Critical Care Quality Control Center (China-NCCQC). The data source was the National Clinical Improvement System collected by the China-NCCQC, which is the official national department that regulates ICU quality control in China. Informed consent was obtained from every hospital. Hospitals with patients of septic shock caused by intestinal perforation admitted in ICUs < 30/year or with incomplete data were excluded from this study. At last, 463 hospitals were involved. The data were collected between January 1, 2018 and December 31, 2018.

In this study, the monitoring indicators were evaluated according to the Chinese clinical quality control indicators for critical care medicine (2015 Edition)^5^. Monitoring indicators included the proportion of ICU patient bed occupancy to total inpatient bed occupancy, the proportion of ICU patients with acute physiology and chronic health evaluation (APACHE) II score ≥ 15, the microbiology detection rate before antibiotic use. The definition of the proportion of ICU patient bed occupancy to total inpatient bed occupancy is (days of ICU bed occupancy by patients)/(days of hospital bed occupancy by patients during the same period). This indicator reflects the proportion and hospital course of ICU patients among all hospitalized patients. The definition of the proportion of ICU patients with acute physiology and chronic health evaluation (APACHE) II score ≥ 15 is (no. of patients with APACHE II score ≥ 15 during the first 24 h in the ICU)/(patients admitted to the ICU during the same period). In general, patients with APACHE II score ≥ 15 are severe patients. This indicator reflects the severity of illness of patients admitted to ICU. The definition of the microbiology detection rate before antibiotic use is (no. of patients with microbiology detection before antibiotics)/(no. of patients who received antibiotics during the same period). This indicator reflects the normative use of antibiotics in ICU. All data of monitoring indicator of participating hospitals and patients were list in Table 1 and S1 Table.

**Table 1:** Information of quality indicators.

Rates (n%)	Number of hospital	Number of patients
Proportion of ICU patient bed occupancy to total inpatient bed occupancy		
< 1	93 (20.09)	1491 (14.46)
1~	127 (27.43)	3019 (29.28)
1.5~	73 (15.77)	1419 (13.76)
2~	71 (15.33)	1481 (14.36)
2.5	99 (21.38)	2900 (28.13)
Proportion of ICU patients with APACHE II score ≥ 15		
< 50	157 (33.91)	4170 (40.45)
50~	57 (12.31)	1295 (12.56)
60~	79 (17.06)	1441 (13.98)
70~	82 (17.71)	1675 (16.25)
80~	88 (19.01)	1729 (16.77)
Microbiology detection rate before antibiotic use		
< 60	103 (22.25)	2136 (20.72)
60~	36 (7.78)	603 (5.85)
70~	54 (11.66)	1180 (11.45)
80~	71 (15.33)	1821 (17.66)
90~	199 (42.98)	4570 (44.33)

ICU, intensive care unit; APACHE, acute physiology and chronic health evaluation.

The outcomes included hospital stays, hospitalization costs, complications, and mortality. The complications included acute respiratory distress syndrome (ARDS) and acute kidney injury (AKI). Based on data obtained from this survey, we will analyze effects of quality control on septic shock caused by intestinal perforation in China.

The authors are accountable for all aspects of the work in ensuring that questions related to the accuracy or integrity of any part of the work are appropriately investigated and resolved. The study was conducted in accordance with the Declaration of Helsinki (as revised in 2013). The trial protocol was approved by the Central Institutional Review Board at Peking Union Medical College Hospital (NO.: S-K1297). The need for written informed consent was waived by the Central Institutional Review Board at Peking Union Medical College Hospital for this retrospective analysis.

The datasets supporting the conclusions of this article are included within the article and its supplementary information files.

### Statistical analysis

Statistical analysis was performed using SPSS software, version 16.0 (Chicago, IL, USA). According to the common clinical tangent points, the relevant indicators of medical quality were converted into categorical variables, and the composition of hospitals and patients in different categories is described in the form of frequency. According to the days of hospital stay, the hospitalized patients were divided into four groups: 1–10 days, 11–20 days, 21–30 days and ≥ 31 days. According to costs, the hospitalized patients were divided into four groups: < 30 thousand yuan (RMB), 30–59.9 thousand yuan (RMB), 60–89.9 thousand yuan (RMB), ≥ 90 thousand yuan (RMB). The Cochran-Armitage trend test was used to determine whether there was a linear trend in medical quality and septic shock caused by intestinal perforation. All of the statistical tests were two-tailed, and a *p* < 0.05 was considered to be statistically significant.

### Ethics approval and consent to participate

The study was conducted in accordance with the *Declaration of Helsinki* (as revised in 2013). The trial protocol was approved by the Central Institutional Review Board at Peking Union Medical College Hospital (NO.: S-K1297). The need for written informed consent was waived by the Central Institutional Review Board at Peking Union Medical College Hospital for this retrospective analysis. The authors are accountable for all aspects of the work in ensuring that questions related to the accuracy or integrity of any part of the work are appropriately investigated and resolved.

## Results

### Association between clinical quality control indicators and hospital stays.

For patients with septic shock caused by intestinal perforation, about a third of hospitals have hospital stays of more than 20 days. Increasing of the proportion of ICU patient bed occupancy to total inpatient bed occupancy increased hospital stays of patients with septic shock caused by intestinal perforation (*p* < 0.05). The proportion of ICU patients with APACHE II score ≥ 15 and the microbiology detection rate before antibiotic use were not associated with hospital stays in patients with septic shock caused by intestinal perforation (Table 2).

**Table 2:** Relationship between quality control indicators and hospital stay.

Rates (n%)	1–10 days	11–20 days	21–30 days	≥ 31 days	*P* for trend
Proportion of ICU patient bed occupancy to total inpatient bed occupancy					0.02
< 1	36.22	36.75	16.03	11.00	
1~	36.73	35.11	15.01	13.15	
1.5~	34.18	36.36	15.72	13.74	
2~	32.82	34.37	16.81	16.00	
2.5	36.21	34.62	15.07	14.10	
Proportion of ICU patients with APACHE II score ≥ 15					0.99
< 50	36.50	34.77	14.75	13.98	
50~	36.22	34.36	14.67	14.75	
60~	31.85	36.57	18.25	13.32	
70~	37.37	34.57	14.51	13.55	
80~	34.36	36.78	16.77	12.09	
Microbiology detection rate before antibiotic use					< 0.05
< 60	36.75	35.53	14.37	13.34	
60~	38.47	34.66	14.59	12.27	
70~	35.42	37.63	15.42	11.53	
80~	35.86	35.09	15.82	13.23	
90~	34.62	34.70	16.11	14.57	

ICU, intensive care unit; APACHE, acute physiology and chronic health evaluation.

### Association between clinical quality control indicators and hospitalization costs

In the treatment of septic shock caused by intestinal perforation, about a quarter of hospitals spend more than 90 thousand yuan (RMB) per person. Increasing of the proportion of ICU patient bed occupancy to total inpatient bed occupancy increased the costs of patients with septic shock caused by intestinal perforation (*p* < 0.05). Increasing of the proportion of ICU patients with APACHE II score ≥ 15 decreased the costs of patients with septic shock caused by intestinal perforation (*p* < 0.05). The microbiology detection rate before antibiotic use was not associated with costs in patients with septic shock caused by intestinal perforation (Table 3).

**Table 3:** Relationship between quality control indicators and costs.

Rates (n%)	< 30 (RMB) (thousand yuan)	30–59.9 (RMB) (thousand yuan)	60–89.9 (RMB) (thousand yuan)	≥ 90 (RMB) (thousand yuan)	*P* for trend
Proportion of ICU patient bed occupancy to total inpatient bed occupancy					< 0.05
< 1	29.38	32.93	16.30	21.40	
1~	28.22	32.23	17.09	22.46	
1.5~	22.55	33.69	17.90	25.86	
2~	22.48	31.53	18.77	27.21	
2.5	23.14	29.45	17.45	29.97	
Proportion of ICU patients with APACHE II score ≥ 15					< 0.05
< 50	22.71	29.54	18.18	29.57	
50 ~	27.26	33.28	16.83	22.63	
60 ~	23.46	32.20	18.04	26.30	
70 ~	28.30	32.54	16.48	22.69	
80 ~	29.03	34.18	16.48	20.30	
Microbiology detection rate before antibiotic use					0.34
< 60	25.19	32.21	17.32	25.28	
60~	28.69	29.68	14.59	27.03	
70~	26.19	31.78	15.25	26.78	
80~	25.81	32.34	17.74	24.11	
90~	24.60	31.33	18.29	25.78	

ICU, intensive care unit; APACHE, acute physiology and chronic health evaluation.

### Association between clinical quality control indicators and complications

When the proportion of ICU patient bed occupancy to total inpatient bed occupancy was greater than or equal to 2, the incidence of ARDS and AKI increased significantly. Increasing of the proportion of ICU patient bed occupancy to total inpatient bed occupancy increased the incidence of complications (ARDS, AKI) in patients with septic shock caused by intestinal perforation (*p* < 0.05). The proportion of ICU patients with APACHE II score ≥ 15 was not associated with the incidence of complications (ARDS, AKI) in patients with septic shock caused by intestinal perforation. The microbiology detection rate before antibiotic use was not associated with the incidence of AKI in patients with septic shock caused by intestinal perforation. When the microbiology detection rate before antibiotic use was greater than or equal to 90, the incidence of ARDS increased significantly. The increase of microbiology detection rate before antibiotic use increased the incidence of ARDS in patients with septic shock caused by intestinal perforation (*p* < 0.05) (Table 4).

**Table 4:** Relationship between quality control indexes and acute organ failure.

Rates (n%)	ARDS	*P* for trend	AKI	*P* for trend
Proportion of ICU patient bed occupancy to total inpatient bed occupancy		< 0.05		< 0.05
< 1	1.88		7.85	
1~	1.66		8.91	
1.5~	1.83		8.39	
2~	4.59		12.49	
2.5	3.28		11.10	
Proportion of ICU patients with APACHE II score ≥ 15		0.48		0.09
< 50	2.49		10.89	
50~	2.47		9.03	
60~	2.78		8.05	
70~	2.45		8.78	
80~	2.89		10.3	
Microbiology detection rate before antibiotic use		< 0.05		0.15
< 60	1.97		9.83	
60~	2.16		8.29	
70~	2.12		10.34	
80~	2.09		8.57	
90~	3.26		10.37	

ICU, intensive care unit; APACHE, acute physiology and chronic health evaluation; ARDS, acute respiratory distress syndrome; AKI, acute kidney injury.

### Association between clinical quality control indicators and mortality

The proportion of ICU patient bed occupancy to total inpatient bed occupancy, the proportion of ICU patients with APACHE II score ≥ 15, and the microbiology detection rate before antibiotic use had no correlation with mortality of the patients with septic shock caused by intestinal perforation (Table 5).

**Table 5:** Relationship between quality control indicators and mortality.

Rates (n%)	Mortality	*P* for trend
Proportion of ICU patient bed occupancy to total inpatient bed occupancy		< 0.05
< 1	12.41	
1~	13.58	
1.5~	10.85	
2~	13.23	
2.5	11.10	
Proportion of ICU patients with APACHE II score ≥ 15		0.40
< 50	12.25	
50~	10.97	
60~	12.49	
70~	12.78	
80~	12.72	
Microbiology detection rate before antibiotic use		0.40
< 60	12.50	
60~	12.44	
70~	12.20	
80~	10.93	
90~	12.74	

ICU, intensive care unit; APACHE, acute physiology and chronic health evaluation.

## Discussion

Increasing of the proportion of ICU patient bed occupancy to total inpatient bed occupancy increased hospital stays, incidence of ARDS and AKI and costs of the patients with septic shock caused by intestinal perforation. The Proportion of ICU patients with APACHE II score ≥ 15 was not associated with hospital stays and incidence of ARDS and AKI. Increasing of the Proportion of ICU patients with APACHE II score ≥ 15 decreased the costs of patients with septic shock caused by intestinal perforation. The microbiology detection rate before antibiotic use was not associated with hospital stays, incidence of AKI and costs of patients with septic shock caused by intestinal perforation. Surprisingly, the increase of microbiology detection rate before antibiotic use increased the incidence of ARDS in patients with septic shock caused by intestinal perforation. The above three indicators had no correlation with mortality of the patients with septic shock caused by intestinal perforation.

Septic shock is characterized by long hospital stay, high costs, many complications and high mortality^6^. Intestinal perforation is a common cause of septic shock^7^. The treatment of this septic shock is extremely difficult due to the complex structure of the abdominal cavity, which makes it difficult to thoroughly drainage and adequate source control^8,9^. In clinical practice, factors such as the timing of intervention for intestinal perforation, the solution of the focus of infection, and the intensity of supportive care are clearly correlated with the treatment effect of septic shock caused by intestinal perforation, and these factors are affected by quality control management. Quality control construction is an important means to improve the level of diagnosis and treatment in ICU^10,11^. Our previous studies shown that quality indicators influencing compliance with the surviving sepsis campaign guidelines in China are the predicted mortality rate and the standardized mortality rate^12^, deep vein thrombosis prophylaxis rate, unplanned extubation rate, reintubation rate within 48 h, and rate of unplanned ICU admission^13^. The compliance with the surviving sepsis campaign guidelines in tertiary hospitals was significantly higher than in secondary hospitals^14^. In order to improve the therapeutic level of septic shock caused by intestinal perforation, we designed this survey to specifically investigate effects of quality control on this type of septic shock.

Higher the proportion of ICU patient bed occupancy to total inpatient bed occupancy means greater the proportion of patients admitted to ICU in the whole hospital^5^. In our survey, the proportion of ICU patient bed occupancy to total inpatient bed occupancy was positively correlated with the increase of hospital stays, incidence of ARDS and AKI and costs of the patients with septic shock caused by intestinal perforation. The main reason for above phenomenon may be high proportion of ICU patients out of total inpatient bed occupancy makes it difficult for ICU to obtain treatment help from other departments of hospital (such as surgical support, puncture support, infectious disease specialist support). As an old Chinese saying goes, the accumulation of water is not thick, and its negative boat is weak.

The Proportion of ICU patients with APACHE II score ≥ 15 reflects the severity of patients admitted to ICU^5^. The Proportion of ICU patients with APACHE II score ≥ 15 was not associated with hospital stays and incidence of ARDS and AKI. Increasing of the Proportion of ICU patients with APACHE II score ≥ 15 decreased the costs of patients with septic shock caused by intestinal perforation. The above results suggest that ICU treatment will not increase hospital stays, incidence of complications, and mortality of severe patients (patients with APACHE II score ≥ 15). On the contrary, with the accumulation of treatment experience, the costs of ICU treatment will decrease. Therefore, ICU admission of severe patients should be encouraged to promote the professionalization of severe patient management.

The microbiology detection rate before antibiotic use reflects the specimen submission status in ICU^5^. In our study, the microbiology detection rate before antibiotic use was not associated with hospital stays, incidence of AKI and costs of patients with septic shock caused by intestinal perforation. This may be because the infection focus of patients with intestinal perforation is the intestinal content and the pathogen types are complex, often including gram-positive cocci, gram-negative bacilli, and even fungi^15,16^. The initial antibiotic selection of septic shock caused by intestinal perforation often covers almost all bacteria, and even fungi^17^. At the same time, the proportion of drug-resistant infections is low in intestinal perforation. Due to the above factors. the correlation between therapeutic effects and microbiology detection rate decreases. Surprisingly, the increase of microbiology detection rate before antibiotic use increased the incidence of ARDS in patients with septic shock caused by intestinal perforation. This may be because repeated collection of sputum specimens increased the incidence of ventilator-associated pneumonia and damage of respiratory mucosa, and eventually increased the incidence of ARDS. This phenomenon suggests that it is not advisable to collect sputum specimens too frequently for patients without pneumonia.

In our survey, the proportion of ICU patient bed occupancy to total inpatient bed occupancy, the Proportion of ICU patients with APACHE II score ≥ 15 and the microbiology detection rate before antibiotic use were not associated with mortality of the patients with septic shock caused by intestinal perforation. The above phenomenon may be related to the fact that major factors affecting mortality of the patients with septic shock caused by intestinal perforation, such as compliance with surviving sepsis campaign guidelines^18,19^, were not included in the study.

There are some limitations in our study. First, only three quality control indicators were included in this study, which may cause some bias to the analysis results. Second, since only one year's of data was included in this study, the effects of quality control on septic shock caused by intestinal perforation could not be analyzed dynamically.

## Conclusions

On the one hand, the number of ICU patients admitted should be controlled to reduce the proportion of ICU patients out of total inpatient bed occupancy, and on the other hand, the number of severe patients (patients with APACHE II score ≥ 15) admitted to ICU should be increased to improve the proportion of patients with APACHE II score ≥ 15 in the ICU, so that ICU can focus more on the treatment of severe patients and promote the professionalization of severe patient management. It is not advisable to collect sputum specimens too frequently for patients without pneumonia.

## Supplementary Information

Supplementary Table 1.

## Data Availability

The datasets supporting the conclusions of this article are included within the article and its supplementary information files.

## Abbreviations

ICU: Intensive care unit
QICC Program: Quality Improvement of Critical Care Program
China-NCCQC: China National Critical Care Quality Control Center
APACHE: Acute physiology and chronic health evaluation
ARDS: Acute respiratory distress syndrome
AKI: Acute kidney injury

## References

[1] Howell MD, Davis AM (2017). Management of sepsis and septic shock. JAMA.

[2] Nikravan S, Song P, Bughrara N, Diaz-Gomez JL (2020). Focused ultrasonography for septic shock resuscitation. Curr. Opin. Crit. Care.

[3] Evans L, Rhodes A, Alhazzani W (2021). Surviving sepsis campaign: International guidelines for management of sepsis and septic shock 2021. Intensive Care Med..

[4] Simsek D, Ozgen G (2021). Recurrent sigmoid volvulus: Cause of colon perforation, sepsis, and fetal death. J. Obstet. Gynaecol. Res..

[5] He H, Ma X, Su L (2020). Effects of a national quality improvement program on ICUs in China: A controlled pre-post cohort study in 586 hospitals. Crit. Care.

[6] Armstrong BA, Betzold RD, May AK (2017). Sepsis and septic shock strategies. Surg. Clin. N. Am..

[7] Clements TW, Tolonen M, Ball CG, Kirkpatrick AW (2021). Secondary peritonitis and intra-abdominal sepsis: An increasingly global disease in search of better systemic therapies. Scand. J. Surg..

[8] Hecker A, Reichert M, Reuss CJ (2019). Intra-abdominal sepsis: New definitions and current clinical standards. Langenbecks Arch. Surg..

[9] Muresan MG, Balmos IA, Badea I, Santini A (2018). Abdominal sepsis: An update. J. Crit. Care Med. (Targu Mures)..

[10] Kumar L, Dominic M, Rajan S, Singh S (2017). Impact of modified quality control checklist on protocol adherence and outcomes in a post-surgical Intensive Care Unit. Indian J. Anaesth..

[11] Sampson BG, Wilson SR, Finnis ME (2018). A quality control study of the adherence to recommended physiological targets for the management of brain-dead organ donors in South Australian Intensive Care units. Prog. Transplant..

[12] Wang L, Ma X, He H (2021). Analysis of structure indicators influencing 3-h and 6-h compliance with the surviving sepsis campaign guidelines in China: A systematic review. Eur. J. Med. Res..

[13] Wang L, Ma XD, He HW (2021). Analysis of factors influencing 3-and 6-h compliance with the surviving sepsis campaign guidelines based on medical-quality intensive care unit data from China. Chin. Med. J..

[14] Wang L, Ma X, He H (2021). Compliance with the surviving sepsis campaign guideline 1-hour bundle for septic shock in China in 2018. Ann. Transl. Med..

[15] Al Nabhani Z, Dietrich G, Hugot JP, Barreau F (2017). Nod2: The intestinal gate keeper. PLoS Pathog..

[16] Kandasamy S, Vlasova AN, Fischer DD (2017). Unraveling the differences between gram-positive and gram-negative probiotics in modulating protective immunity to enteric infections. Front. Immunol..

[17] Snydman DR (2012). Empiric antibiotic selection strategies for healthcare-associated pneumonia, intra-abdominal infections, and catheter-associated bacteremia. J. Hosp. Med..

[18] Mukherjee V, Evans L (2017). Implementation of the surviving sepsis campaign guidelines. Curr. Opin. Crit. Care.

[19] Rodrigues-Santos G, de Magalhaes-Barbosa MC, Raymundo CE, Lima-Setta F, da Cunha A, Prata-Barbosa A (2021). Improvement of 1st-hour bundle compliance and sepsis mortality in pediatrics after the implementation of the surviving sepsis campaign guidelines. J. Pediatr..

