# Supplementary material for: Association between quality control and outcomes of septic shock caused by intestinal perforation in China: a cross-sectional study
Source: Sci Rep. 2023 Feb 27;13:3373. doi: 10.1038/s41598-023-30551-w (PMC9971201; doi:10.1038/s41598-023-30551-w)
Supplement: Supplementary file 1 — Supplementary Table 1. [file 41598_2023_30551_MOESM1_ESM.docx]

**Supply Table 1 Basic information of patients**

|  | N | n% |
| --- | --- | --- |
| total | 10310 | 100 |
| gender |  |  |
| male | 6809 | 66.04 |
| female | 3501 | 33.96 |
| age (year) |  |  |
| <30 | 365 | 3.54 |
| 30-39 | 393 | 3.81 |
| 40-49 | 794 | 7.70 |
| 50-59 | 1384 | 13.42 |
| 60-69 | 2402 | 23.30 |
| 70-79 | 2836 | 27.51 |
| 80-89 | 1917 | 18.59 |
| ≥90 | 219 | 2.12 |
| basic diseases |  |  |
| coronary heart disease | 739 | 7.17 |
| diabetes | 803 | 7.79 |
| chronic obstructive pulmoriary disease | 645 | 6.26 |
| chronic kidney disease | 237 | 2.30 |
| chronic liver failure | 462 | 4.48 |
| lupus | 30 | 0.29 |
| hospital stay (day) |  |  |
| 1-10 | 3670 | 35.60 |
| 11-20 | 3637 | 35.28 |
| 21-30 | 1601 | 15.53 |
| ≥31 | 1402 | 13.60 |
| costs  (RMB) (thousand yuan) |  |  |
| <30 | 2614 | 25.35 |
| 30-59.9 | 3263 | 31.65 |
| 60-89.9 | 1797 | 17.43 |
| ≥90 | 2636 | 25.57 |
| death | 1267 | 12.29 |
